# Insight into the Effect of TDMs on the Tribological Behaviors of the Ionic Liquid Composite Films

**DOI:** 10.3390/ma13010191

**Published:** 2020-01-02

**Authors:** Ya’e Qi, Ling Zhang, Yongxia Wang

**Affiliations:** 1 College of Chemistry and Chemical Engineering, Hexi University, Key Laboratory of Hexi Corridor Resources Utilization of Gansu, Zhangye 734000, China; qiyaezhu@163.com; 2 Shenzhen CONE Tech. Co., Ltd. 51&52 Building, Software Town of Shenzhen Universiade Longgong, Shenzhen 518100, China; 3 College of Environmental Science and Engineering, Donghua University, 2999 Ren’min North Road, Shanghai 201620, China

**Keywords:** ionic liquid film, MoS_2_ nanosheet, graphene, friction, anti-wear

## Abstract

Ionic liquid (IL) combined with 2D materials has evoked considerable attention in the field of lubrication applications because of their speical structure and outstanding lubrication properties. However, the ambiguous effect of the 2D materials on the friction and anti-wear properties of the IL needs futher study. Here, we have obtained two families of IL composite films with additives of MoS${}_{2}$ and graphene via a combined process of spin-coated and curing, and the distinction of the effects of two additives on the tribological performance of the IL films was studied. The friction tests showed that the friction coefficient and anti-wear life of the IL films were greatly enhanced after the addition of MoS${}_{2}$ or graphene, which could be attributed to the improved load-carrying capacity and the second lubrication phase. Under a low addition content, graphene had more advantages to reduce the friction of the films, and MoS${}_{2}$ was more beneficial to the tribological properties with the additional content increased. The films with low friction and good anti-wear properties may be valuable for the rational design of lubrication films for the practical engineering applications.

## 1. Introduction

Industrial equipment and devices are in constant motion, but the severe wear and interfacial adhesion have become great challenges for the moving and contact parts in the mechanical moving equipment [1,2,3]. Good lubrication has become a crucial issue for improving performance reliability and reducing the energy consumption of the moving parts, and a solid lubricant on the contact surface of moving parts has become an important choice. Since the initial report in 2001 [4], ionic liquids (IL) with special configuration and outstanding tribological performance have evoked a great deal of attention in the area of lubricating applications [5,6,7,8,9,10].

Based on the current reports, it has been supposed that the IL film could be used as a kind of important lubricant to reduce the friction and improve the anti-wear for the moving parts [6,11,12]. However, under the boundary lubricated condition, the insufficient load-carrying capacity and anti-wear performance of the IL films greatly limited their wide applications [13]. For the past few years, a lot of additives have been reported to enhance the tribological performance of IL with low friction coefficient and improved anti-wear ability [14,15,16,17]. Two-dimensional materials (TDMs), such as graphene and molybdenum disulfide (MoS${}_{2}$), with outstanding anti-wear and ultralow friction coefficient, have become attractive solid lubricants to be used as additives to enhance a lubricating property of mechanical components [18,19,20,21].

The microtribological results of the lubricating composite film based on IL and graphene demonstrate that the friction coefficient and anti-wear ability of the IL film decrease with the graphene content increased, and the enhanced tribological performance could be ascribed to the synergic effect of IL and graphene in the composite films [22]. IL composite films with the functionalized graphene which could be evenly dispersed in IL without overlapping exhibited an improved load-carrying capacity and anti-wear compared with the pure IL film, attributing to the synergic effect between the additives and IL acted as the load-carrying and lubricating phase, respectively [14]. Moreover, it has been suggested that the composite film of IL/functionalized-graphene expands the application scope of IL lubrication film and shows an important application value in the moving parts [23]. In our previous work, it has been found that the MoS${}_{2}$ nanosheets as additives could greatly enhance the anti-wear properties of the IL films, attributing to the synergistic effect of the IL and MoS${}_{2}$ [24].

Combining the present reports, the appropriate amount of TDMs (graphene and MoS${}_{2}$) as additives show a great potential to enhance the tribological performance of IL film, and the corresponding mechanism of additives affecting the reduced friction and improving anti-wear properties is mainly ascribed to the improved load-carrying capacity of the composite films. Comparing the lubrication effect of graphene and MoS${}_{2}$, it has been considered that MoS${}_{2}$ nanosheets possessed an advantage over graphene, as the latter performed relatively higher and less stable tribological properties [25]. However, there are few works about the difference of two kinds of additives (graphene and MoS${}_{2}$) on the tribological properties of the IL-based films. Meanwhile, although the tribological performance and lubrication mechanism of the IL films have already been thoroughly described in other literature, the effect of lubricating additives on friction and anti-wear properties of IL based films still needs to be studied further.

Herein, with the aim to study the effect of 2D materials (TDMs) as additives on the tribological properties of the IL composite films, two kinds of nanosheets (graphene and MoS${}_{2}$) have been added in the IL composite films. The friction and anti-wear properties of series of the IL films with a different added amount of TDMs were studied, and the effect of the two lubricating additives on tribological properties of IL films were analyzed. The comparison of the effect of graphene and MoS${}_{2}$ acting as additives on the tribological performance of the IL films may be valuable for the rational design of IL-based lubricants in the practical lubrication applications.

## 2. Materials and Methods

### 2.1. Preparation of the IL Composite Films

The liquid exfoliation process of the MoS${}_{2}$ nanosheets has been described in the previous work [24]. Typically, a certain amount of the bulk MoS${}_{2}$ (100 mg, 15 μm, Nanjing MKNANO Tech. Co., Ltd, Nanjing, China) and DMF (C${}_{3}$H${}_{7}$NO, 30 mL, Aladdin Reagents Co., Ltd, Shanghai, China) were exposed to ultrasound treatment for 4 h, and then the obtained MoS${}_{2}$ suspensions were centrifuged at a speed of 2000 rpm for 20 min. The upper centrifuged suspension was extracted under 12,000 rpm for 30 min, and, finally, a few-layer MoS${}_{2}$ nanosheets were cleared with ethanol and deionized water 3 times, respectively.

**Figure 1:**
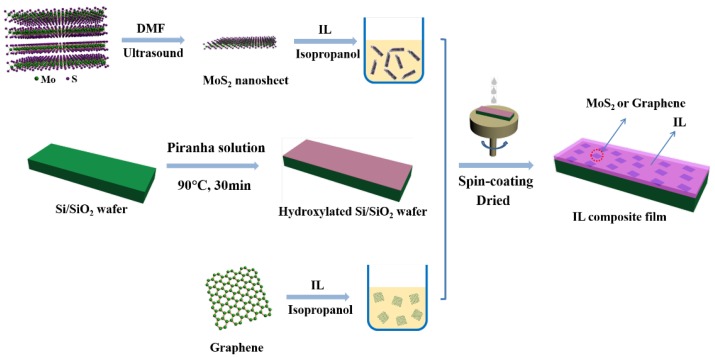
Preparation of the IL/MoS${}_{2}$ or IL/graphene composite films via a spin-coating process.

The formation of the IL ([BMIM]BF${}_{4}$, Sinopharm Chemical Reagent Co., Ltd, Ningbo, China) composite films added with MoS${}_{2}$ and graphene (Nanjing XFNANO Tech. Co., Ltd, Nanjing, China) was shown in Figure 1. The typical process was listed as follows: a different amount of obtained MoS${}_{2}$ nanosheets and graphene were dispersed into the isopropyl alcohol solution of IL (5 mg/mL) under continuous ultrasonication, and the ultimate mass ratios of MoS${}_{2}$ or graphene in IL were 0.1 wt%, 0.3 wt%, 0.5 wt%, 1 wt%, and 2 wt%, respectively. The IL composite films were coated on the hydroxylated Si/SiO${}_{2}$ wafers (obtained by immersing into the freshly prepared piranha solution 3(98% H${}_{2}$SO${}_{4}$)/1(30% H${}_{2}$O${}_{2}$) at 90 ${}^{\circ }$C for 30 min.) by means of spin coating, and were heated at 120 ${}^{\circ }$C for 30 min in N${}_{2}$. Finally, the obtained composite films of IL with MoS${}_{2}$ were named IL/0.1M, IL/0.3M, IL/0.5M, IL/1M, IL/2M, respectively, and the films of IL with graphene were IL/0.1G, IL/0.3G, IL/0.5G, IL/1G, IL/2G, respectively.

### 2.2. Characterization

A Transmission Electron Microscope (TEM, JEM-2100F) was used to observe the morphology of the MoS${}_{2}$ and graphene. The thickness and morphology of the MoS${}_{2}$ nanosheets and graphene were obtained via Atomic Force Mcroscope (AFM, Asylum Research MFP-3D). The microstructure of the MoS${}_{2}$ nanosheets and graphene was studied by Raman spectra (inVia-Reflex, excitation wavelength: 532 nm).

### 2.3. Tribological Tests

The tribological tests of a series of IL composite films were carried out with a ball-disk friction tester (HSR-2M). Under the frequency of 4.2 Hz and reciprocating distance of 5 mm, the steel balls (GCr15) with a diameter of 6 mm were used to run against the IL composite films with a load of 100 g. Each pair was tested thrice to take the average value. Optical microscopy was used to observe the worn scar of the steel balls and composite films, and the information of the chemical component on the worn scar of the ball sliding with the IL/0.3M and IL/0.3G was determined via XPS (escalab 250xi, Thermo Fisher Scientific).

## 3. Results and Discussion

**Figure 2:**
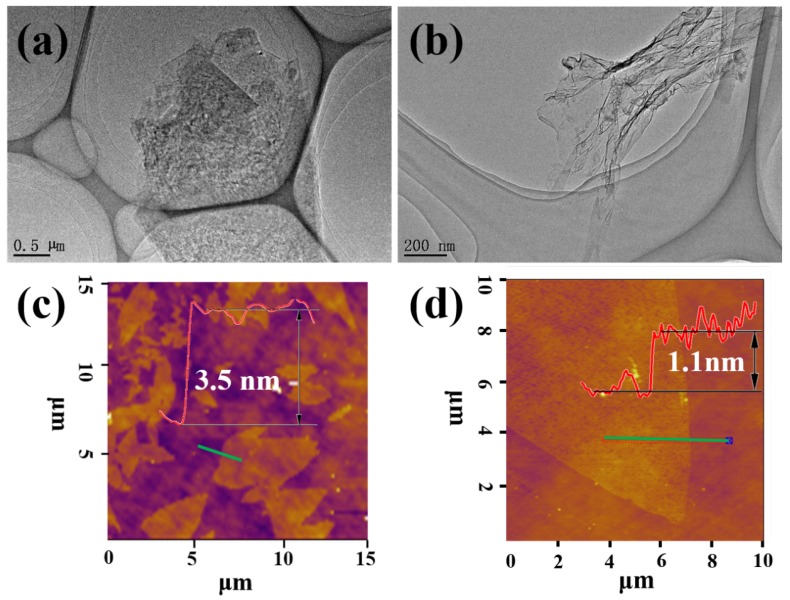
TEM and AFM images of the MoS${}_{2}$ (**a**,**c**) and graphene (**b**,**d**).

The morphology of the MoS${}_{2}$ and graphene are observed via TEM, and the typical images are shown in Figure 2a,b. From the TEM images, it can be seen that both MoS${}_{2}$ and graphene have a nanosheet structure. AFM is applied to confirm the thickness of exfoliated MoS${}_{2}$ nanosheets and graphene, and the typical Raman spectra of the MoS${}_{2}$ nanosheets and graphene are shown in Figure S1.

For MoS${}_{2}$ shown in Figure 2c, it has a step height of individual layers of 3.5 nm, which is compatible with the 0.62 nm for a single layer of the MoS${}_{2}$ crystal [26], and thus the layer number of the exfoliated MoS${}_{2}$ nanosheets is about 5. The above results show that the MoS${}_{2}$ produced by exfoliated process has a few-layers nanosheet structure, which is consistent with the previous reports for exfoliated MoS${}_{2}$. From the AFM image of the graphene shown in Figure 2d, it can be seen that a layer structure of graphene is formed. The cross-sectional height reveals that the thickness of the graphene is 1.1 nm, and, according to the previous reported thickness of 0.34 nm [27], the layer number is about 3.

**Figure 3:**
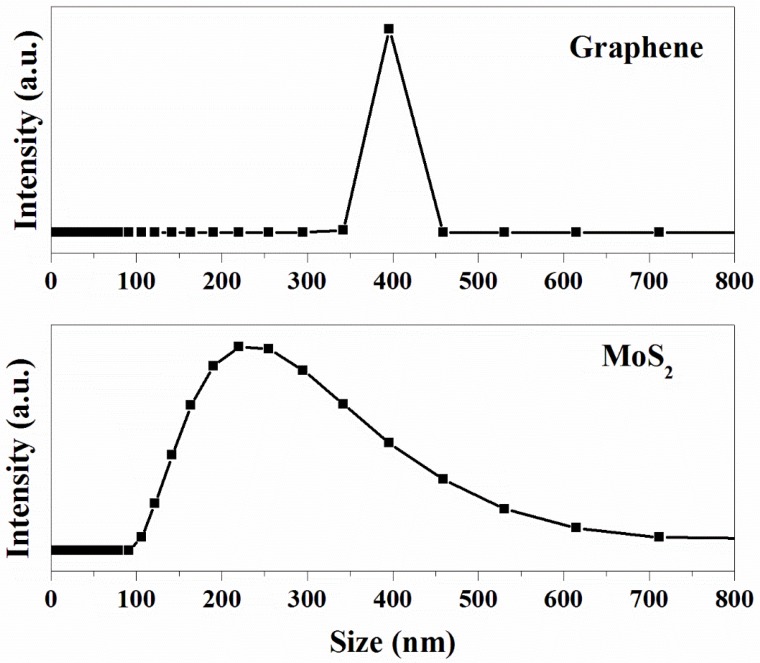
Average size distribution of the MoS${}_{2}$ nanosheets and graphene.

The average size of the MoS${}_{2}$ and graphene are shown in Figure 3, from which it can be seen that the size of the MoS${}_{2}$ nanosheets is about 220 nm. The average size of graphene is 395 nm, which is much higher than that of the MoS${}_{2}$. The results show that the exfoliated MoS${}_{2}$ nanosheets have a relatively small particle size.

**Figure 4:**
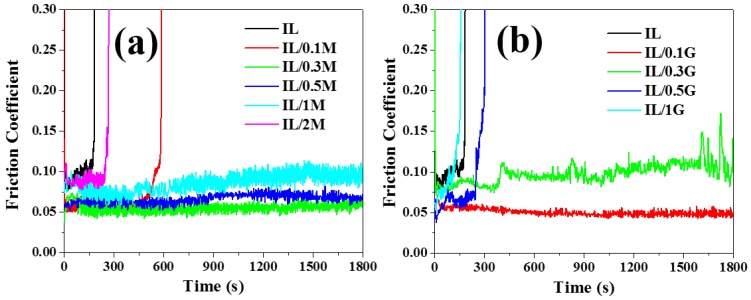
Friction coefficient as a function of sliding time for the IL films with different amounts of MoS${}_{2}$ nanosheets (**a**) and graphene (**b**).

The friction and anti-wear properties for the series of the IL composite films with MoS${}_{2}$ and graphene are tested by a friction tester under the load of 100 g. Figure 4a,b give the relation between the friction coefficient with sliding time of the IL composite films with MoS${}_{2}$ and graphene, respectively, from which, it can be seen that the pure IL film exhibits a relatively high friction coefficient up to 0.1, and, more importantly, it shows an extreme short anti-wear life, ascribing to the poor load-carrying capacity. For the composite films with the addition of MoS${}_{2}$, they show improved anti-wear life and reduced friction coefficient. Combining the results reported in the previous work, the IL composite film with MoS${}_{2}$ content increased to 0.3 wt%, and the anti-wear life of the composite film of IL/0.3M increases 10 times. The friction coefficient of the composite film increases gradually with the additional content of MoS${}_{2}$ increased, and, for the composite film with 2 wt% MoS${}_{2}$, the anti-wear life dramatically reduces.

The results of the friction test for the IL composite films with a different additional content of graphene are shown in Figure 4b, and it can be seen that a relatively low content of graphene (0.1 wt%) can greatly reduce the friction coefficient while prolonging the anti-wear life. However, with the content of graphene increased to 0.3 wt%, the friction coefficient of the composite films increases, and the anti-wear life of the composite films with 0.5 wt% exhibits a sharp decrease.

Based on the above results, appropriate content MoS${}_{2}$ nanosheets and graphene can greatly enhance the friction and anti-wear properties of the IL films. For the IL composite films with a low content of the additives (0.1 wt%), graphene has more advantages for reducing the friction coefficient and improving the anti-wear life. With the increase of the content of the additives (0.3 wt%), MoS${}_{2}$ is more beneficial to the tribological properties.

**Figure 5:**
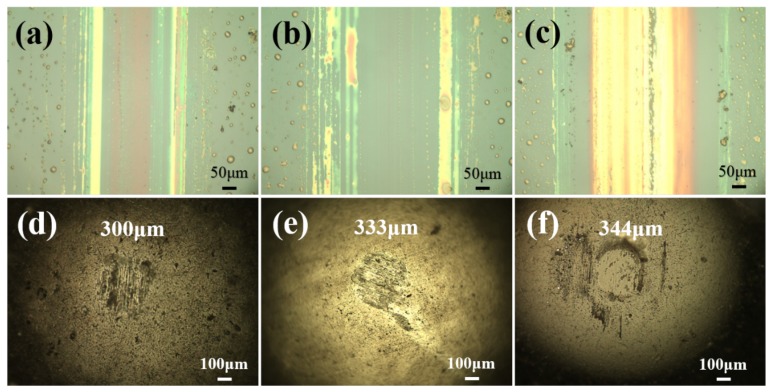
The worn morphology of the IL/M composite films and steel balls: IL/0.3M (**a**,**d**), IL/0.5M (**b**,**e**), and IL/1M (**c**,**f**).

The surface morphology of the worn scar for the series of the IL composite films with MoS${}_{2}$ are given in Figure 5, and it can be observed that the IL/0.3M film shows the smallest wear width. With the increase of additional content of the MoS${}_{2}$, the wear width increases gradually. For the steel ball sliding with the composite films, the wear scar diameter exhibits a similarity with the trend of wear width of the composite films. The IL composite film with high content of MoS${}_{2}$ shows poor wear resistance, indicating that the MoS${}_{2}$ may play a negative impact on the tribological performance of the IL films.

**Figure 6:**
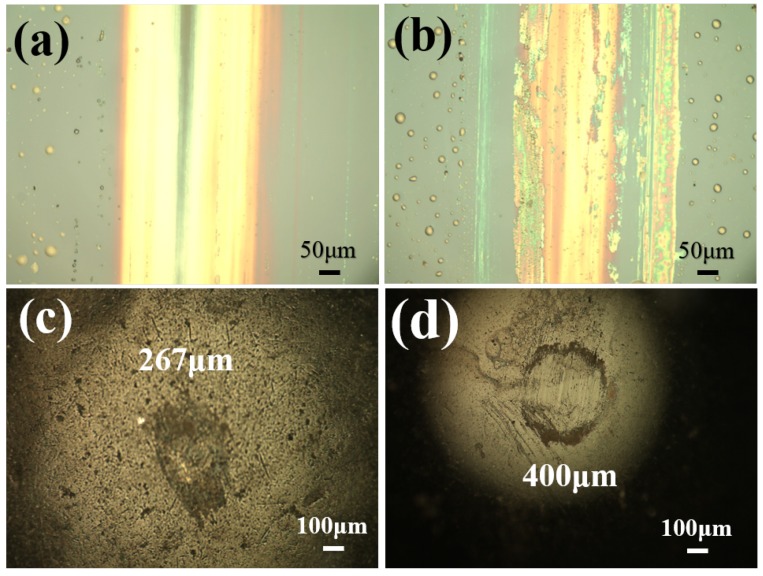
The morphology of the worn scar for the IL/G composite films and corresponding steel balls: IL/0.1G (**a**,**c**) and IL/0.3G (**b**,**d**).

The worn morphology of the composite films of IL with different content of graphene is observed via optical microscope and shown in Figure 6. It can be seen that the wear scar of IL/0.1G is much smaller than that of the composite film of IL/0.3G. Compared to the results of the wear diameter of the steel ball sliding with IL composite films with 0.3 wt% MoS${}_{2}$ and graphene, the IL/0.3M shows a smaller value than that of IL/0.3G, indicating that, under the same content of the additives, MoS${}_{2}$ has more advantages for improving the anti-wear ability of the films. Meanwhile, it can be easily observed that the worn scar of the steel ball is coated by a layer of transferred materials, and, at the same time, a large amount of wear debris gathered on the side of the wear scar of the IL/0.3G.

In most cases, wear scar diameter (WSD) has been used to estimate to wear behaviors of the lubricants, and, from the WSD, the wear volume (*V*) can be determined by the relations as follows [28,29] by:

(1) $$V=\left(\frac{\pi h}{6}\right)\left(\frac{3d^{2}}{4}+h^{2}\right),$$

(2) $$h=r-sqrt(r^{2}-\frac{d^{2}}{4}),$$

where *d* and *r* are the wear scar diameter and the radius of the steel ball, respectively.

Based on the above equations and the measured wear scar diameter (*d*) of the steel ball, *V* is calculated and the corresponding values are listed in Table 1. For the IL composite with MoS${}_{2}$, the IL/0.3M composite film shows the smallest wear volume, and the wear volume of the IL/M gradually becomes large with the increase of the content of MoS${}_{2}$, indicating that the high addition content of MoS${}_{2}$ exhibits a adverse effect on the tribological performance of the IL films. For the IL film with 0.1 wt% graphene (IL/0.1G), it shows a relatively small value of *V*. With the increase of graphene content to 0.3 wt%, the wear volume of the composite film greatly increases, and it shows a five-fold larger than that of the IL with 0.1 wt%.

**Figure 7:**
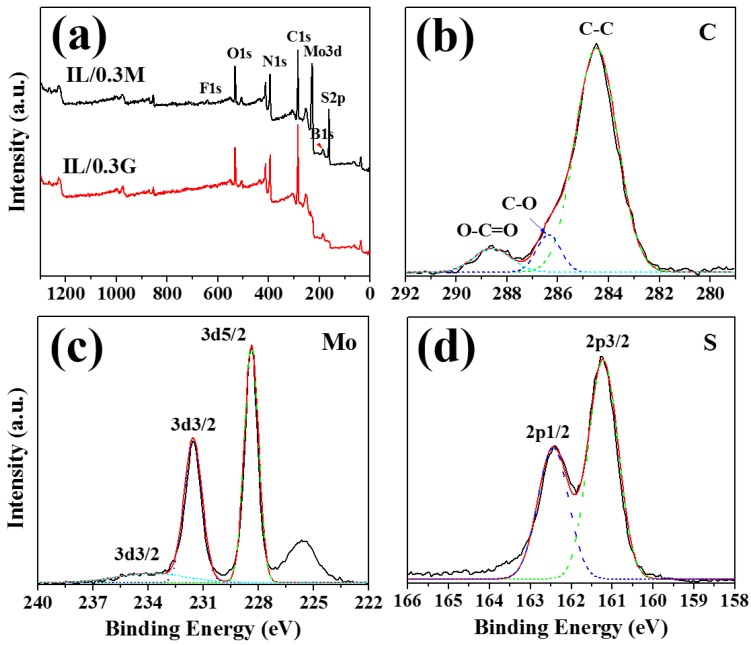
XPS survey spectra (**a**) of the wear scar of the steel balls sliding with IL/0.3M and IL/0.3G, C1s for ball-IL/0.3G (**b**), and Mo3d (**c**), S2p (**d**) for ball-IL/0.3M.

The chemical component on the worn scar of the steel balls sliding with the IL composite films is studied via XPS, in order to explore the lubrication mechanism of the 2D nanosheets as additives for the IL films. From the survey spectrum of the two composite films shown in Figure 7a, it can be seen that two obvious peaks existed at 399.8 eV and 194 eV, corresponding to N1s and B1s peaks, can be attributed to the N and B in IL (C${}_{8}$H${}_{15}$N${}_{2}$BF${}_{4}$). The above result indicates that the element of the IL could be transferred to the contact surface of the steel ball during the sliding with IL composite films. Additionally, the Mo3d and S2p peaks at 232.3 eV and 161.8 eV can be detected in the wear scar, indicating that the products of Mo and S in the IL/0.3M films transferred to the steel ball.

The detailed information of the C1 peaks are given in Figure 7b, and the Gaussian method is used to fit the corresponding peaks. The main peak centered at 284.5 eV is assigned to C in graphene, indicating that graphene would be transferred to the wear scar of the steel ball. In the meantime, the weak signal of C-O and O-C=O peaks at 286.4 eV and 288.6 eV, respectively, is detected, which may be attributed to the oxidation of the graphene during sliding, or the oxycarbides on the steel ball exposed to air. For the Mo 3d, two peaks at 232.8 eV and 228.8 eV are assigned to Mo 3d3/2 and 3d5/2 of MoS${}_{2}$, and a weak signal of MoO${}_{3}$ at 234 eV is detected, indicating that the transferred MoS${}_{2}$ is oxidized lightly. For the S2p, S 2p1/2 and 2p3/2 can be seen at 163.8 eV and 161.3 eV, attributing to S in MoS${}_{2}$. From the above results, it can be concluded that transferred IL and MoS${}_{2}$ or graphene exist on the worn surface of the steel balls sliding with the IL/0.3M or IL/0.3G.

**Figure 8:**
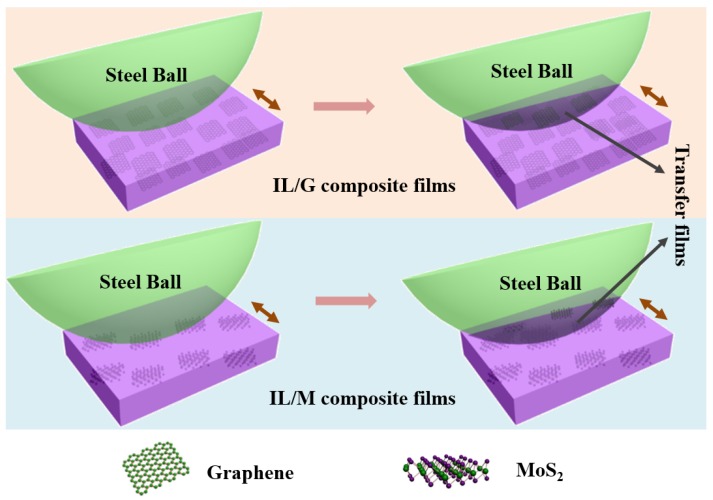
Schematic illustration of the tribological mechanism of the IL composite films.

Based on the results of the tribological tests and the characterization of the worn surface of the steel balls sliding with series of the IL composite films, the greatly enhanced friction and anti-wear properties of the IL/M films can be attributed to the improved load-carrying capacity and the formed transfer films on the worn scar of the steel ball (Figure 8). Under a given load, the worse tribological properties of pure IL film may be ascribed to the poor load-carrying capacity of the films. For the IL composite films with 2D nanosheets as additives, they can greatly improve the load-carrying capacity of the composite film and thus give it longer anti-wear life than the IL film. Additionally, MoS${}_{2}$ or graphene as an effective solid lubricant may also act as the second lubricating phase to further enhance the friction and anti-wear properties of the composite films. The improved load-carrying capacity and second lubricating phase give the composite films a low friction coefficient and prolonged anti-wear life.

For the IL composite films with a low content of additives, graphene has more advantages to improve the tribological properties of the films, and MoS${}_{2}$ is more beneficial to the tribological properties with the increase of the addition content. Graphene is one-atom thick carbon arranged in a honeycomb structure, and MoS${}_{2}$ has a trilayer structure with one Mo atomic layer sandwiched between two S atomic layers [30,31]. Under the same content of the additives, specific surface area of graphene is much higher than that of the MoS${}_{2}$ nanosheets, and thus a greater amount (volume) of graphene exists in the same volume of IL composite films. The addition of nanosheet in the IL composite films plays a disadvantageous effect on the film continuity, and excess graphene has a negative impact on the structure and continuity of the IL composite films. The morphology of the IL composite films with a different amount of MoS${}_{2}$ or graphene is shown in Figure S2, from which it can be seen that the surface morphology of the IL film becomes rough with the content of graphene increased to 1 wt%. The deterioration of the film continuity would give a poor load-carrying capacity and eventually lead to the degradation of the tribological performance of the IL composite films (as shown in Figure S3).

## 4. Conclusions

A series of IL based composite films composed of IL and TDMs (MoS${}_{2}$ or graphene) were obtained and the tribological properties of the composite films were studied. The friction coefficient and anti-wear life of the IL films were greatly improved with MoS${}_{2}$ or graphene as additives, and the enhanced tribological properties of the composite films were ascribed to the improved load-carrying capacity and the second lubrication phase. Graphene has more advantages for reducing friction of the films with a low content of additives, and, with the increase of the additional content, MoS${}_{2}$ is more beneficial to the tribological properties of the composite films. Excess addition of the additives would cause the deterioration of the film continuity and a poor load-carrying capacity, and eventually lead to the degradation of the tribological performance of the IL composite films.

## Figures and Tables

**Table 1:** Wear volume (*V*) of the steel ball sliding with different IL composite films.

	IL/0.3M	IL/0.5M	IL/1M	IL/0.1G	IL/0.3G
*r* (mm)	3	3	3	3	3
*d* (mm)	0.3	0.333	0.344	0.267	0.4
*h* (um)	375	462	493	297	667
*V* ($10^{-6}$ mm${}^{3}$)	133	201	229	83	419

## Supplementary Materials

The following are available online at https://www.mdpi.com/1996-1944/13/1/191/s1, Figure S1. Raman spectra of the MoS${}_{2}$ nanosheets and graphene; Figure S2. Morphology of the IL composite films observed by optical microscope: (a) IL, (b) IL/0.1M, (a) IL/1M, (a) IL/0.1G, (a) IL/1G; Figure S3. The wear process of the nonuniformity IL composite with excess additives.

## References

[1] Izabela S., Michael C., Robert W.C. (2008). Recent advances in single-asperity nanotribology. J. Phys. D Appl. Phys..

[2] Bhushan B., Israelachvili J.N., Landman U. (1995). Nanotribology: Friction, wear and lubrication at the atomic scale. Nature.

[3] Bhushan B. (2017). Nanotribology and nanomechanics of MEMS/NEMS and bioMEMS/BioNEMS materials and devices. Nanotribology and Nanomechanics: An Introduction.

[4] Ye C., Liu W., Chen Y., Yu L. (2001). Room-temperature ionic liquids: A novel versatile lubricant. Chem. Commun..

[5] Bonhote P., Dias A.P., Papageorgiou N., Kalyanasundaram K., Grätzel M. (1996). Hydrophobic, highly conductive ambient-temperature molten salts. Inorg. Chem..

[6] Yu B., Zhou F., Mu Z., Liang Y., Liu W. (2006). Tribological properties of ultra-thin ionic liquid films on single-crystal silicon wafers with functionalized surfaces. Tribol. Intern..

[7] Somers A., Howlett P., MacFarlane D., Forsyth M. (2013). A review of ionic liquid lubricants. Lubricants.

[8] Mo Y., Yu B., Zhao W., Bai M. (2008). Microtribological properties of molecularly thin carboxylic acid functionalized imidazolium ionic liquid film on single-crystal silicon. Appl. Surf. Sci..

[9] Bhushan B., Palacio M., Kinzig B. (2008). AFM-based nanotribological and electrical characterization of ultrathin wear-resistant ionic liquid films. J. Colloid Interf. Sci..

[10] Lertola A.C., Wang B., Li L. (2018). Understanding the friction of nanometer-thick fluorinated ionic liquids. Ind. Eng. Chem. Res..

[11] Zhao W., Pu J., Yu Q., Zeng Z., Wu X., Xue Q. (2013). A novel strategy to enhance micro/nano-tribological properties of DLC film by combining micro-pattern and thin ionic liquids film. Colloids Surf. A Physicochem. Eng. Asp..

[12] Zhao W., Wang Y., Wang L., Bai M., Xue Q. (2010). Influence of heat treatment on the micro/nano-tribological properties of ultra-thin ionic liquid films on silicon. Colloids Surf. A Physicochem. Eng. Asp..

[13] Zhang L., Pu J., Wang L., Xue Q. (2014). Frictional dependence of graphene and carbon nanotube in diamond-like carbon/ionic liquids hybrid films in vacuum. Carbon.

[14] Pu J., Wan S., Zhao W., Mo Y., Zhang X., Wang L., Xue Q. (2011). Preparation and tribological study of functionalized graphene-IL nanocomposite ultrathin lubrication films on Si substrates. J. Phys. Chem. C.

[15] Pu J., Huang D., Wang L., Xue Q. (2010). Tribology study of dual-layer ultrathin ionic liquid films with bonded phase: influences of the self-assembled underlayer. Colloids Surf. A Physicochem. Eng. Asp..

[16] Zhao W., Zhu M., Mo Y., Bai M. (2009). Effect of anion on micro/nano-tribological properties of ultra-thin imidazolium ionic liquid films on silicon wafer. Colloids Surf. A Physicochem. Eng. Asp..

[17] Lv M., Han F., Wang Q., Wang T., Liang Y. (2017). The structure properties and tribological behavior of the ionic liquid-polyimide composite films under high-vacuum environment. High Perform. Polym..

[18] Rasheed A.K., Khalid M., Rashmi W., Gupta T.C.S.M., Chan A. (2016). Graphene based nanofluids and nanolubricants-review of recent developments. Renew. Sustain. Energy Rev..

[19] Xu Y., Peng Y., Dearn K.D., Zheng X., Yao L., Hu X. (2015). Synergistic lubricating behaviors of graphene and MoS_2_ dispersed in esterified bio-oil for steel/steel contact. Wear.

[20] Zhang W., Cao Y., Tian P., Guo F., Tian Y., Zheng W., Ji X., Liu J. (2016). Soluble exfoliated two-dimensional nanosheets as excellent aqueous lubricants. ACS Appl. Mater. Int..

[21] Wu X., Gong K., Zhao G., Lou W., Wang X., Liu W. (2018). Surface modification of MoS_2_ nanosheets as effective lubricant additives for reducing friction and wear in poly-*α*-olefin. Ind. Eng. Chem. Res..

[22] Zhao W., Zeng Z., Peng S., Wu X., Xue Q., Chen J. (2013). Fabrication and investigation the microtribological behaviors of ionic liquid-graphene composite films. Tribol. T.

[23] Palacio M., Bhushan B. (2008). Ultrathin wear-resistant ionic liquid films for novel MEMS/NEMS applications. Adv. Mater..

[24] Wang Y., Cao X.A., Lang H., Zeng X., Chen B., Chen R., Peng Y. (2018). Enhanced Tribological properties of composite films based on ionic liquids with MoS_2_ nanosheets as additives. N. J. Chem..

[25] Zhao J., He Y., Wang Y., Wang W., Yan L., Luo J. (2016). An investigation on the tribological properties of multilayer graphene and MoS_2_ nanosheets as additives used in hydraulic applications. Tribol. Int..

[26] Splendiani A., Sun L., Zhang Y., Li T., Kim J., Chim C.Y., Galli G., Wang F. (2010). Emerging photoluminescence in monolayer MoS_2_. Nano Lett..

[27] Stankovich S., Dikin D.A., Piner R.D., Kohlhaas K.A., Kleinhammes A., Jia Y., Wu Y., Nguyen S.T., Ruoff R.S. (2007). Synthesis of graphene-based nanosheets via chemical reduction of exfoliated graphite oxide. Carbon.

[28] Liang S., Shen Z., Yi M., Liu L., Zhang X., Ma S. (2016). In-situ exfoliated graphene for high-performance water-based lubricants. Carbon.

[29] Berman D., Erdemir A., Sumant A.V. (2013). Reduced wear and friction enabled by graphene layers on sliding steel surfaces in dry nitrogen. Carbon.

[30] Chua C.K., Pumera M. (2013). Covalent chemistry on graphene. Chem. Soc. Rev..

[31] Jiang J.W. (2015). Graphene versus MoS_2_: A short review. Front. Phys..

