# Supplementary material for: Insight into the Effect of TDMs on the Tribological Behaviors of the Ionic Liquid Composite Films
Source: Materials (Basel). 2020 Jan 2;13(1):191. doi: 10.3390/ma13010191 (PMC6981414; doi:10.3390/ma13010191)
Supplement: Supplementary file 1 [file materials-13-00191-s001.pdf]

materials

MDPI

Article

Insight into the Effect of TDMs on the Tribological Behaviors of the Ionic Liquid Composite Films

Yaé Qi [1], Ling Zhang [2] and Yongxia Wang [3,*]

[1] College of Chemistry and Chemical Engineering, Hexi University, Key Laboratory of Hexi Corridor Resources Utilization of Gansu, Zhangye 734000, China; qiyaezhu@163.com
[2] Shenzhen CONE Tech. Co., Ltd. 51&52 Building, Software Town of Shenzhen Universiade Longgong, Shenzhen 518100, China; zhangling@chinacone.com
[3] College of Environmental Science and Engineering, Donghua University, 2999 Ren'min North Road, Shanghai 201620, China, China
* Correspondence: wyx912@dhu.edu.cn

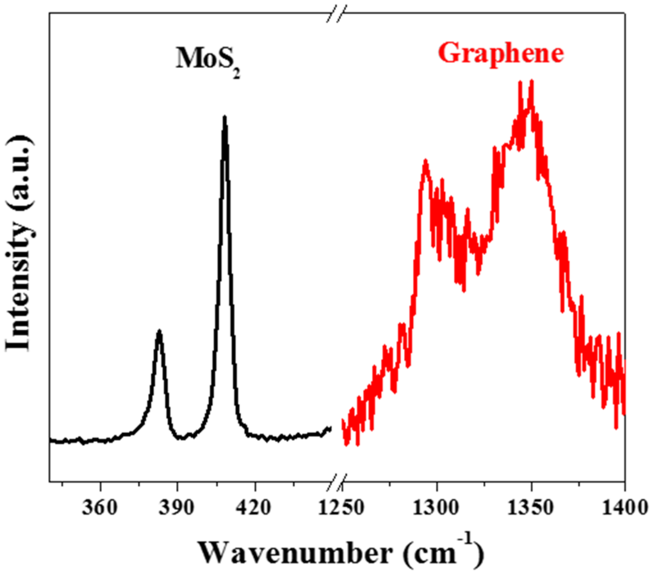

Figure S1. Raman spectra of the MoS_2 nanosheets and graphene.

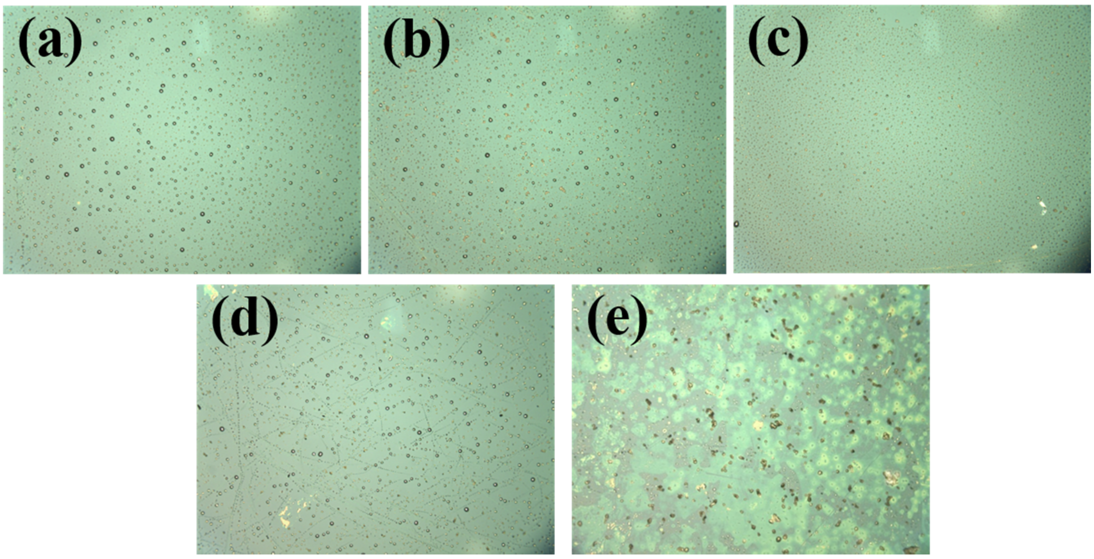

Figure S2. Morphology of the IL composite films observed by optical microscope: (**a**) IL, (**b**) IL/0.1M, (**c**) IL/1M, (**d**) IL/0.1G, (**e**) IL/1G.

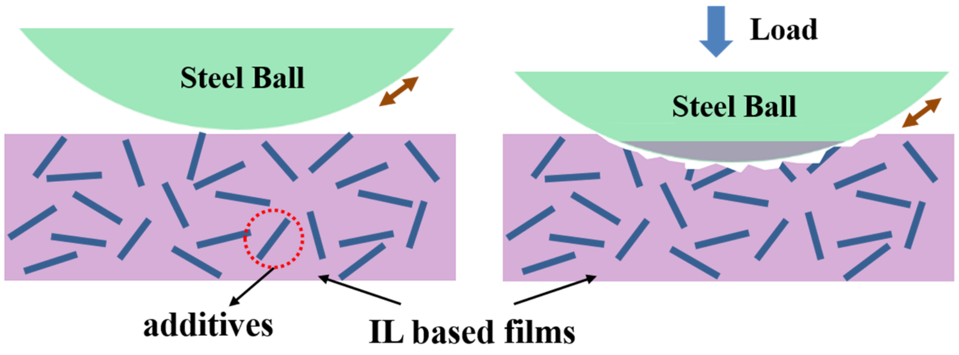

Figure S3. The wear process of the nonuniformity IL composite with excess additives.

© 2020 by the authors. Licensee MDPI, Basel, Switzerland. This article is an open access article distributed under the terms and conditions of the Creative Commons Attribution (CC BY) license (http://creativecommons.org/licenses/by/4.0/).
